# Practical Skills of Rhythmic Gymnastics Judges

**DOI:** 10.2478/hukin-2013-0087

**Published:** 2013-12-31

**Authors:** Maria A. Fernandez-Villarino, Marta Bobo-Arce, Elena Sierra-Palmeiro

**Affiliations:** 1 Faculty of Education and Sport Sciences, University of Vigo, Spain.; 2 Faculty of Sport Science and Physical Education, University of Coruña, Spain.

**Keywords:** Practical knowledge, sport judges, rhythmic gymnastics

## Abstract

The aim of this study was to analyze the practical skills of rhythmic gymnastics judges and to identify how their degree and experience influence the assessment of these skills. Sixty one rhythmic gymnastics judges participated in the study. A questionnaire was used for data collection. This tool was composed of 28 questions and divided into six categories: identification, experience, initial training, continuing education, skills and training needs. The results suggest that the most valued skills are those related to the sport’s technical parameters and the ability to adapt to any level of competition with self-confidence and self-assuredness. Significant differences were found regarding the variables for: the ability to communicate (p = 0.002) and for the ability to observe, identify and register performance (p = 0.005). The results showed that experience was not a decisive factor in assessing skills. This study thus presents evidence that rhythmic gymnastics judges must implement and optimise a set of skills that contribute to the effectiveness of the assessment process. These findings might help in the design of programs and training models that contribute to effective professional development.

## Introduction

Compared to other sports in which performance is objectively measured (e.g., by goals, time, or distance travelled), gymnasts’ scores in rhythm gymnastics are obtained solely from judges’ evaluations made during competition. These evaluations include quantitative objective measures such as the number and difficulty level of body and apparatus performance elements, in addition to qualitative subjective measures, such as aesthetic impression and harmony of music and movement.

MacMahon and Mildenhall (2012) suggest that professional experience and practical skills may influence the subjective evaluation of gymnasts’ performances. However, there are few studies that have investigated the characteristics, knowledge and abilities that rhythmic gymnastics judges must possess and that may determine the efficacy of their judgments.

Traditionally, most research regarding sports judging and refereeing has been performed in the area of sports psychology (Weimberg and Richardson, 1990). These studies have covered various topics, such as the following: (1) stress (Goldsmith and Williams, 1992), (2) decision-making (Plessner and Betsch, 2001), (3) perception (Phillips, 1985) and (4) personality traits (Antonelli and Salvini, 1975). With specific reference to gymnastics, the list can be expanded by the following topics: (5) psychological skills (Persichini, 1985; Teryokhina, 1997), (6) judgment biases (Popovic, 2000; Boen et al., 2008), (7) expert judgment (Plessner and Schallies, 2005) and (8) assessment instruments and procedures (Palomero, 1998). Nevertheless, there is little research that investigates the definition and validation of judges’ and referees’ knowledge of sports in general (Renon and Falcon, 2007; Ghasemi et al., 2009) and of rhythmic gymnastics in particular (Cabrera, 1998).

Understanding the factors that influence the effectiveness of a judge’s actions may be useful in designing programs and training models that foster effective professional development (Mascarenhas et al., 2005). In such an undertaking, it is necessary to identify specific knowledge that is focused and contextualised (Bromme, 1988), while adjusting the theoretical frame for practical reality to avoid training models that are based solely on technical rationalism (Schön, 1984).

The aim of this study was twofold: (1) to analyze the practical skills of rhythmic gymnastics judges and (2) to identify how judges’ qualification levels and experience influenced the assessment of these skills. Thus, two hypotheses were proposed: (1) skills most valued by the judges would be those psychological abilities that were developed through “practical knowledge”; and (2) the judges with international qualifications and higher levels of experience would place greater value on those skills specifically related to objectification of their judgments.

## Material and Methods

### Participants

The participants in this study were 61 rhythmic gymnastics judges. All the subjects were female (age: 35.21, SD± 10.16). The inclusion criteria for participants in this study were as follows: (1) to be officially licensed by the Royal Spanish Gymnastics Federation (RSGF) and (2) to participate in official competitions of the RSGF as a member of the panel of judges. Thirty-eight (62.3%) of the participants in the study held the title of a national judge, and 23 (37.7%) held the title of an international judge. With respect to experience as judges, 13 (21.3%) had fewer than five years’ experience and 48 (78.7%) had five or more years of experience.

### Measures

The measurement instrument was a semi-structured questionnaire consisting of 28 questions, which included 18 closed-ended and 10 open-ended questions. Construction of the questionnaire was carried out in the following phases (Cohen and Manion, 2000): (a) a review of the relevant literature on the subject matter; (b) development of the initial questionnaire that was then tested on an expert; (c) development of a second questionnaire that was tested in a pilot study with autonomic level judges; and (d) preparation of the final questionnaire that was structured along the dimensions of identification, experience, initial training, continuing education, skills and training needs.

The analyzed variables included the following: (1) Degree of qualification, which was divided into the two categories of national judges (indicating that the judges are qualified to participate in regional and national level competitions) and international judges (indicating that the judges are qualified to participate in international competitions, in addition to regional and national competitions); (2) Experience, which was divided into two categories, following Burden (1990), that were defined by whether judges had less than or more than five years of experience in judging at the national championship level; (3) Ability to judge any level of competition, (4) Self-confidence and self-assuredness, (5) Ability to control pressure activation, (6) Ability to communicate, (7) Observation, interpretation and recording skill, (8) Ability to differentiate the level of performance, (9) Ability to objectify emotional perceptions; (10) Aesthetic appreciation; (11) Knowledge of technical parameters; and (12) Knowledge of artistic and musical parameters. Internal consistency for these variables was assessed by Cronbach’s Alpha, which was determined to have a value of α = 0.65.

### Procedures

The data were collected during four official gymnastics national championships in 2011 that were organized by the Royal Spanish Gymnastics Federation (RSGF). We contacted all the participants during the judges draw meeting, at which time the subjects were recruited. When they volunteered to participate in the study, they were fully informed about the aim and design procedures of the research. At the end of the meeting, the completed questionnaires were collected. The study was performed in accordance with the ethical standards laid down in the 1964 Declaration of Helsinki, with the consent of the National Gymnastics Judges.

### Statistical Analysis

The results are presented as the mean and standard error (SE). The distribution of the sample was checked by means of the Shapiro-Wilk Test. An unpaired *t*-test for continuous variables was used for comparing the two factors, i.e. the level of qualification (National vs International), and years of experience as a judge (a dummy variable with a value of (0) = under five years or (1) = five years or more). All statistical analysis was performed using SPSS for Windows, version 20.0 (SPSS Inc. IBM, USA). In all analysis, a significance level of *p* < 0.05 was considered.

## Results

Table 1 shows the descriptive data for each variable for all the judges participating in the study; the data suggest that the most valued skills include the ability to judge any level of competition, self-confidence and self-assuredness, and knowledge of technical parameters. Less valued skills are: ability to differentiate the level of performance, ability to communicate, and aesthetic appreciation of the exercise being scored.

Table 2 shows descriptive data for the variables classified according to the level of the judges (national or international). The data suggest significant differences regarding the variables for the ability to communicate (*t* = 3.12, *p* = 0.002) and observational, interpretive and recording skills (*t* = 2.87, *p* = 0.005). However, no differences were found for variables such as the ability to objectify emotional perceptions, aesthetic appreciation, and knowledge of artistic and musical parameters

Table 3 presents the values of the variables categorized according to judges’ experience and shows that no significant differences were found for any of the analyzed variables.

## Discussion

This study analyzed the practical skills of rhythmic gymnastics judges and how they value their abilities, according to their experience and their qualifications level as a judge. The results suggest that the abilities most valued by the judges participating in the study are knowledge of the technical parameters of the sport and the capacity to adjust to any level of competition under self-assuredness and self-confidence circumstances. These results are in agreement with Anshell (1995) and Cabrera (1998) who claim that the key component for efficient judging is knowledge and comprehension of the rules. Additionally, Weinberg and Richardson (1990) and Ittenbach and Eller (1998) indicate that trustworthiness is a key factor when they describe optimum psychological skills that differentiate best judges and referees. Moreover, our results suggest that the ability to communicate is not recognised as a determining variable, which is inconsistent with the findings of Weinberg and Richardson (1990) who found that judges and referees considered this ability to be significant. The finding of our study that judges do not consider communication abilities to be particularly valuable may be understood in the context of the position of rhythmic gymnastics judges, who do not intervene in the sports action and who are forbidden from engaging in communications with gymnasts or coaches during the competition.

During a typical rhythmic gymnastics competition, a judge must evaluate approximately 150 performances that last approximately 1 minute and 30 seconds each (in the case of an individual competition). These exercises are executed by gymnasts of different ages and of different levels. This specific situation suggests that the ability to discriminate performances should be emphasized by the judges participating in our study; as Mascharenhas (2004) explains, higher level referees have greater abilities to discriminate performances. Nonetheless, our findings do not support this position.

Teryokhina (1997) notes that the quality of judging is negatively influenced by the excessive amounts of information presented by a gymnast to a judge during an exercise. Not only is knowledge of the rules critical to the rigor of the process but also a level of experience is necessary to enable the judge to quickly anticipate, identify and register all the actions executed by the gymnast. However, in our study, experience was not considered a key factor for a judge in acquiring these skills. Starkes (2000) notes that experience is related to actual practice and not necessarily to the number of years holding an official license. Thus, the total amount of specific and actual practice should contribute to the qualifications of a professional judge, as occurs in other sports. Along these lines, Catteeuw et al. (2009) obtained positive correlation indices among hours practiced per week, the number of competitions judged and the ability to evaluate a performance.

In addition, our aim was to analyze the relationship between judges’ abilities and the objectification of value judgments. Rhythmic gymnastics judging demands great mental capacity because it combines emotional perception with objective technical evaluation; therefore, the judge must simultaneously appreciate expressive, artistic and technical parameters. However, no significant differences in these abilities were found that would lead us to confirm the initial hypothesis that it is impossible for a judge to simultaneously evaluate objective-technical parameters and subjective-aesthetic parameters.

In other sports and in similar situations in which various types of information must be processed simultaneously, Catteeuw et al. (2009) analyzed different judging roles and proposed creating specific roles within the panel of judges to optimize the assessment of every component to be evaluated.

One of the limits of the current study was the small sample size. Future studies should compare our findings with those of other countries and/or other sports.

Our study is constrained by the absence of prior research with respect to judging sports competitions and the professional skills required to do so. Most prior research has focused on referees, not judges, but these two figures typically are assigned different types of tasks. In addition, prior studies have approached this topic from the perspective of psychology. By contrast, the aim of the current study was to address the “know-how” of judges, which forms the knowledge base of their professional development. Similarly, if the sample were larger and the level of experience were otherwise accounted for, we might have obtained higher rates of significance in the results, which would have allowed us to better identify the skills required in the professional development of rhythmic gymnastics judges.

Future studies should address how psychological and physiological mechanisms can influence the development of the studied variables. Experience measured in years (five years was the benchmark used in this study) should not be the only factor taken into account in determining an expert (Abraham et al., 2006).

In conclusion, this study offers evidence that the rhythmic gymnastics judge must apply and optimize a set of skills that contribute to an effective assessment process, including not only abilities that come from initial training—such as knowledge of the rules—but also abilities that are developed through analysis and reflection during practical experiences. Theoretical knowledge of the rules set in the code of points does not guarantee the ability to interpret and apply them; thus, specific practical training and a continuous evaluation of the judge’s capacity to observe, interpret and identify performance is necessary. Thus, considering this sport’s particular characteristics, we believe that continuous training should be oriented toward the development of specific abilities—mainly those related to the objectification of emotional perception and aesthetic appreciation—which should ensure an assessment of performance that is as consistent and accurate as possible.

## Figures and Tables

**Table 1:** Descriptive values of practical skills for all judges

	All (N=61)		
Variables	Mean	SE	(95%CI)
Qualification	1.38	0.063	(1.25–1.50)
Experience	0.80	0.052	(0.69–0.90)
Ability to Judge any Level of Competition	1.31	0.065	(1.18–1.44)
Self Confidence and Self-assuredness	1.33	0.069	(1.19–1.47)
Control Presure Activation	1.51	0.073	(1.37–1.66)
Ability to Comunicate	1.90	0.091	(1.71–2.08)
Observe, Interpret and Record	1.35	0.062	(1.22–1.47)
Differentiate the Level of Performance	2.03	0.097	(1.83–2.22)
Objectify Emotional Perception	1.78	0.106	(1.57–1.99)
Aesthetic Appreciation	1.81	0.093	(1.62–2.00)
Domain Technical Parametres	1.31	0.065	(1.18–1.44)
Domain Artistic and Musical Parametres	1.46	0.076	(1.31–1.62)

SE: Standard error; CI: Confidence interval.

**Table 2:** Practical skills evaluation based on the level of the judges.

	National (n=38)			International (n=23)			*t*-test	
Variables	Mean	SE	(95%CI)	Mean	SE	(95%CI)	*t*	p
AJC	1.34	0.077	(1.19–1.51)	1.26	0.112	(1.02–1.49)	0.610	0.544
SCS	1.39	0.096	(1.20–16.0)	1.21	0.087	(1.03–1.40)	1.250	0.215
ACP	1.55	0.097	(1.36–1.76)	1.43	0.105	(1.21–1.65)	0.785	0.435
ACT	2.10	0.111	(1.87–2.34)	1.56	0.122	(1.31–1.82)	3.125	0.002
OIR	1.47	0.082	(1.31–1.65)	1.13	0.071	(0.98–1.27)	2.871	0.005
DLP	2.13	0.110	(1.91–2.36)	1.86	0.181	(1.49–2.24)	1.327	0.189
OEP	1.84	0.133	(1.59–2.13)	1.65	0.173	(1.29–2.01)	0.870	0.387
AAA	1.89	0.111	(1.65–2.12)	1.69	0.159	(1.36–2.02)	1.049	0.298
DTP	1.34	0.077	(1.19–1.51)	1.26	0.112	(1.02–1.49)	0.610	0.544
AMP	1.55	0.097	(1.36–1.76)	1.30	0.116	(1.06–1.54)	1.603	0.114

SE: Standard error; CI: Confidence interval; AJC: Ability to judge any level of competition; SCS: Self-confidence and self-assuredness; ACP: Ability to control pressure activation; ACT: Ability to communicate; OIR: Ability to observe, interpreted and record; DLP: Ability to differentiate the level of performance; OEP: Ability to objectify the emotional perception; AAA: aesthetic appreciation; DTP: Domain of technical parameters; AMP: Domain of artistic and musical parameters

**Table 3:** Practical skills evaluation based on the experience of the judges.

	Experience <5 years (n=13)			Experience >= 5 years (n=48)			*t*-test	
Variables	Mean	SE	(95%CI)	Mean	SE	(95%CI)	*t*	p
AJC	1.46	0.143	(1.14–1.77)	1.27	0.071	(1.12–1.41)	1.221	0.226
SCS	1.23	0.121	(0.96–1.49)	1.35	0.081	(1.19–1.51)	−0.729	0.468
ACP	1.30	0.133	(1.01–1.59)	1.56	0.083	(1.39–1.73)	−1.452	0.151
ACT	1.92	0.177	(1.53–2.31)	1.89	0.104	(1.68–2.10)	0.123	0.902
OIR	1.30	0.133	(1.01–1.59)	1.35	0.069	(1.21–1.49)	−0.307	0.759
DLP	2.16	0.207	(1.71–2.62)	2.00	0.111	(1.77–2.22)	0.677	0.500
OEP	1.69	0.237	(1.17–2.20)	1.79	0.118	(1.55–2.03)	−0.382	0.703
AAA	1.84	0.191	(1.42–2.26)	1.81	0.105	(1.59–2.02)	0.148	0.882
DTP	1.23	0.121	(0.96–1.49)	1.33	0.074	(1.18–1.48)	−0.651	0.517
AMP	1.46	0.143	(1.14–1.77)	1.45	0.089	(1.27–1.63)	0.017	0.986

SE: Standard error; CI: Confidence interval; AJC: Ability to judge any level of competition; SCS: Self-confidence and self-assuredness; ACP: Ability to control pressure activation; ACT: Ability to communicate; OIR: Ability to observe, interpreted and record; DLP: Ability to differentiate the level of performance; OEP: Ability to objectify the emotional perception; AAA: aesthetic appreciation; DTP: Domain of technical parameters; AMP: Domain of artistic and musical parameters
